# Radiographic Healing and Observed Complications Following Light-Cured Polymer Immobilization: A Retrospective Cohort Study of 108 Patients

**DOI:** 10.3390/jcm15103709

**Published:** 2026-05-12

**Authors:** Onix Reyes Martínez, James Stavitz, Kenielle Olmeda-Mercado, Viviana Negrón-Rodríguez, Ryan Porcelli

**Affiliations:** 1 Huesos Chicos, Orthopedics for Children, Adolescents, Adults & Sports, San Juan 00674, Puerto Rico; 2 Department of Athletic Training, Kean University, Union, NJ 07083, USA; 3 Department of Biology, University of Puerto Rico at Arecibo, Arecibo Campus, Arecibo 00614, Puerto Rico; 4 School of Medicine, Ponce Health Sciences University, Ponce 00716, Puerto Rico; vnegron23@stu.psm.edu

**Keywords:** fracture fixation, immobilization, fracture healing, pediatric orthopedics, retrospective studies, orthopedic procedures

## Abstract

**Purpose:** Traditional plaster and fiberglass casts remain widely used for fracture immobilization but are associated with recognized challenges, including skin irritation, hygiene limitations, and distress during cast removal, particularly in pediatric populations. Light-cured polymer immobilization (LCPI) systems have been introduced as an alternative method of fracture support. The primary objective of this study was to describe radiographic healing and alignment outcomes among patients treated with an LCPI system. Secondary objectives were to document skin- and device-related events and to identify any unplanned removals or subsequent re-interventions. **Methods:** A 6-month retrospective cohort study was conducted involving 108 consecutive patients treated with an LCPI system between January and June 2025 at a single orthopaedic clinic. Clinical and radiographic records were reviewed to extract demographic information, injury characteristics, treatment details, immobilization duration, healing outcomes, alignment status, and recorded adverse events. Outcomes were summarized using descriptive statistics. **Results:** Immobilization was applied for 104 fractures (96.3%), three sprains (2.8%), and one elbow dislocation (0.9%). The cohort (76 males, 32 females; mean age: 13.4 years; range: 4–53) demonstrated radiographic union or progression toward union among fracture cases with available follow-up imaging. Mean immobilization duration was 29.2 days (SD: 6.2; range: 10–48). Alignment at device removal was documented as anatomic or near-anatomic in 103 of 104 fractures (99.1%) based on treating clinician assessment (99.1%). Device breakage was documented in 12 cases (11.1%), of which 3 required additional immobilization. Two patients (1.9%) experienced mild cutaneous reactions that resolved with conservative management. No severe device-related complications were documented. **Conclusions:** Healing outcomes and recorded adverse events were consistent with expected clinical patterns for this patient population in this descriptive retrospective cohort of patients treated with an LCPI system. These findings provide descriptive real-world data regarding clinical utilization and short-term outcomes in selected patients. Prospective comparative studies are needed to further define effectiveness, safety, cost considerations, and broader applicability across diverse fracture populations.

## 1. Introduction

Fracture immobilization using plaster of Paris (POP) and fiberglass has been a fundamental component of orthopaedic care for over a century because of its accessibility, low cost, and generally reliable support of fracture healing [1,2,3]. Although widely effective, these materials are associated with a spectrum of documented complications, most commonly minor skin irritation or cast-related discomfort [4,5,6]. Reported adverse events include maceration, localized pressure injury, and thermal irritation during application, while more serious complications such as compartment syndrome are considered uncommon and are more frequently associated with fracture severity, high-energy trauma, or evolving soft tissue swelling rather than immobilization alone [4,5]. In pediatric populations, particularly in minimally displaced fractures, complication rates are low. In a randomized controlled trial of 96 children with distal radius fractures, Boutis et al. reported complication rates of 6.5% in the cast group and 8.0% in the splint group, with no significant difference between treatment approaches [7]. Dadkhah-Tehrani et al. (2022) identified pressure ulcers in 1.7% of immobilized patients and noted that unplanned cast removal occurred in 15.2% of cases, often related to pain, swelling, or cast discomfort rather than structural treatment failure [6]. Some studies have reported complication rates approaching 25%, with the majority classified as minor skin- or hygiene-related events rather than severe medical complications [8,9].

Pediatric casting carries unique risks due to thinner, more sensitive skin prone to the effects of pressure, heat, and moisture [10]. McGraw-Heinrich et al. (2025) noted erythema, maceration, and thermal injury during application and removal [5]. DiPaola et al. (2014) found that 5.3% of casts required unplanned changes (mean 13 days), most commonly due to wetness (47%) or breakage (33%), with 3% of patients developing irritation and two cases of superficial infection requiring antibiotics [11]. DiFazio et al. (2017) demonstrated that targeted padding reduced lower extremity cast complications [8]. Wong et al. (2018) reported that therapeutic play lowered distress during cast removal [12], while Georgiadis et al. (2025) found that virtual reality reduced anxiety in children aged 4–12 [13]. Daşar et al. (2024) further highlighted the psychological challenges associated with cast removal, emphasizing the distress linked to conventional cast-saw procedures [14].

New immobilization options such as hybrid-mesh casts, thermoplastic splints, 3D-printed, and resin-filled casts aim to address the drawbacks of traditional casting. In a randomized trial of 79 children, Ong et al. (2023) found hybrid-mesh casts improved comfort and satisfaction but took longer to remove (4.18 ± 1.25 vs 2.25 ± 0.55 min; *p* < 0.001) and cost more [15]. Al Khudairy et al. (2012) showed that thermoplastic splints maintained alignment and had high satisfaction with few complications but required specialized fabrication [16]. Although 3D-printed orthoses can provide stabilization with improved fit and patient comfort, recent advances in additive manufacturing have enabled the development of customized, lightweight immobilization devices with improved ventilation and patient-specific geometry. However, many designs lack integrated cushioning, and their widespread use remains limited. Skibicki et al. (2022) and van Lieshout et al. (2022) both noted that despite favorable short-term outcomes and low complication rates, 3D printing is hindered by high production costs, long fabrication times, and the need for specialized software, printers, and trained personnel [17,18]. Additional work has highlighted ongoing efforts to improve design efficiency and clinical scalability, although practical and economic barriers to widespread adoption remain [19]. Similar resin-filled, non-cushioned lattice systems that harden through chemical curing within a short arm sleeve have been explored, though limited peer-reviewed evidence describing their clinical performance is currently available.

Light-cured polymer immobilization (LCPI) systems have been introduced as an alternative approach to fracture support with the aim of improving patient comfort, hygiene, and handling characteristics while maintaining adequate mechanical stability. These systems typically consist of a photo-curable polymer lattice combined with padding and an external protective layer, resulting in a lightweight, breathable, and water-resistant immobilization construct that may be removed without the use of a traditional oscillating cast saw. The open-lattice configuration allows visualization of the underlying skin and may facilitate ventilation during immobilization. Early clinical investigations have reported acceptable short-term healing outcomes in selected fracture populations [20]. In a multicenter cohort of 137 distal radius fractures treated with a light-cured polymer system, Bali et al. (2024) reported radiographic union in 100% of cases, with two minor superficial skin infections and no documented loss of reduction [20]. A subsequent narrative review emphasized potential workflow and patient-experience advantages while also highlighting the need for larger real-world evaluations across broader clinical indications [21].

However, published real-world data describing LCPI use across a broader range of clinical indications remain limited. The objective of this retrospective study was to describe radiographic healing patterns and alignment outcomes among consecutive patients managed with an LCPI at a single orthopaedic clinic between 1 January and 30 June 2025. Secondary objectives were to document skin- and device-related events, quantify unplanned removals and subsequent re-interventions, and summarize patterns of immobilization management during routine follow-up.

## 2. Methods

### 2.1. Study Design and Setting

This investigation was conducted as a retrospective observational study at a single orthopaedic specialty clinic (Huesos Chicos Paediatric Orthopaedics and Sports Medicine Clinic, San Juan, Puerto Rico). The study was conducted in accordance with the Strengthening the Reporting of Observational Studies in Epidemiology (STROBE) guidelines (Supplemental Table S1) [22]. Consecutive patients who underwent immobilization using a LCPI system between January and June 2025 were eligible for inclusion. Patients were included if sufficient clinical documentation and radiographic follow-up at or near the time of device removal were available to allow for assessment of healing and alignment outcomes. Patients with premature device removal were included if follow-up clinical and/or radiographic documentation was available.

### 2.2. Patients

**Figure 1:**
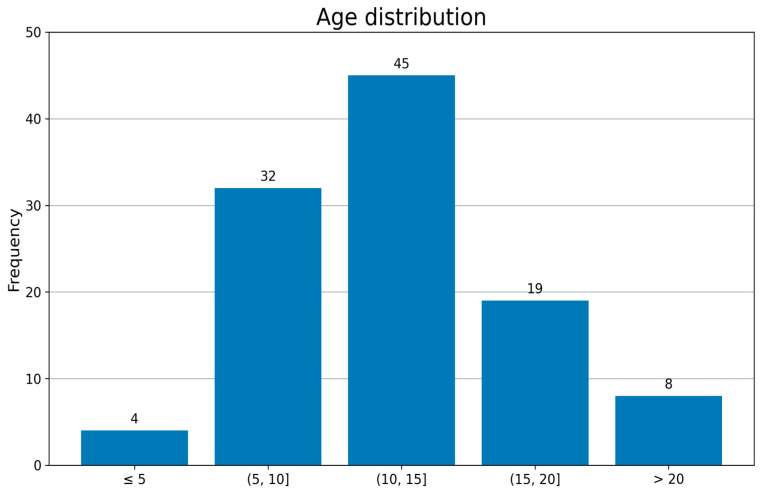
Age distribution of the study cohort (N = 108). Histogram illustrates frequency of immobilization across pediatric, adolescent, and adult age groups.

**Figure 2:**
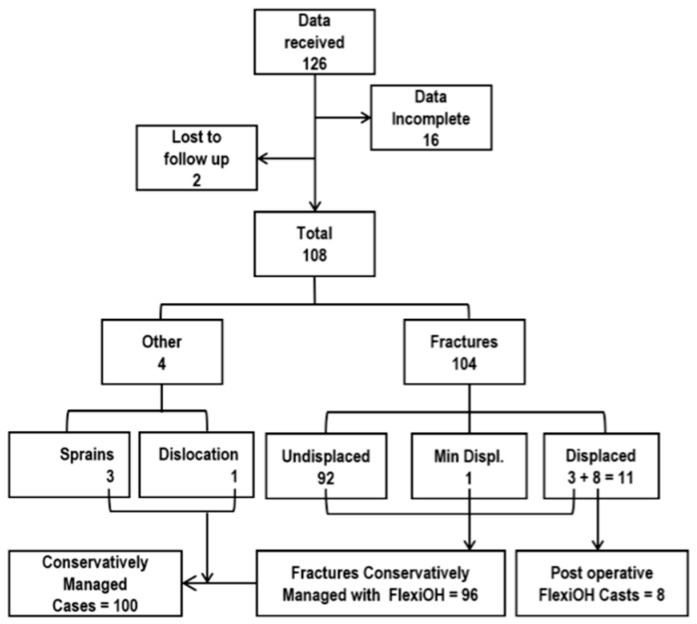
Distribution of fractures, non-fracture injuries, and management subgroups. Categories include conservative fracture management, post-surgical immobilization, sprains, and elbow dislocation.

Clinical records were reviewed for all patients who underwent immobilization using an LCPI system during the study period. A total of 126 patients were identified. Sixteen patients were excluded due to incomplete clinical or radiographic documentation, and two patients were excluded because they were lost to follow-up prior to device removal. The final analytic cohort consisted of 108 patients with complete documentation available for review. The cohort included 76 males and 32 females with a mean age of 13.4 ± 7.8 years (range: 4–53 years), consistent with a predominantly pediatric and adolescent patient population (Table 1; Figure 1). Patients were included if immobilization was applied for any clinical indication and both pre-application and post-removal clinical and radiographic information were documented in the medical record. The distribution of injury types and management pathways is presented in Figure 2.

### 2.3. Data Collection

Clinical data were retrospectively abstracted from the electronic medical record using a standardized data collection template. Extracted information was de-identified prior to review by the investigative team, and no protected health information was included in the analytic dataset. Two investigators independently reviewed the abstracted data for completeness and internal consistency, with discrepancies resolved through discussion with the treating clinical team.

Variables collected included patient demographics, injury characteristics, treatment details, duration of immobilization, and follow-up interval. Radiographic healing status and fracture alignment were determined based on treating clinician interpretation as documented in formal radiology reports and orthopaedic clinic notes recorded at follow-up visits. No independent radiographic re-evaluation was performed as part of this study. Device-related and skin-related events documented in the medical record, including breakage, irritation, unplanned device removal, or need for additional immobilization, were also recorded. Missing data were not imputed.

Additional variables included the clinical indication for immobilization (fracture management, postoperative support, or soft-tissue injury), fracture location and pattern, baseline displacement status, and relevant comorbidities when documented. Details regarding initial management prior to application of the LCPI, including use of splints or conventional casts, were also collected. Information related to device duration, planned versus premature removal, and subsequent immobilization following device removal was recorded when available. Definitive immobilization decisions, including transition from interim management to LCPI, were based on treating clinician judgment and standard clinical practice considerations. The LCPI devices used in this study were obtained as part of routine clinical care and were not donated by the manufacturer for research purposes.

### 2.4. Light-Cured Polymer Immobilization System

**Figure 3:**
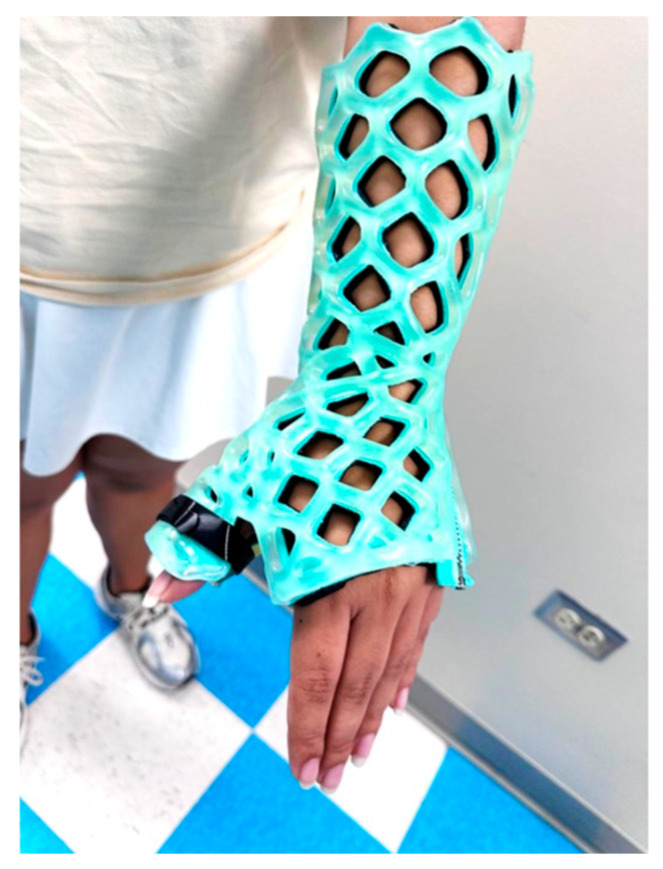
Thumb Spica LCPI immobilizer applied to the upper extremity. The image demonstrates the external lattice structure, silicone cover, and thumb containment position.

**Figure 4:**
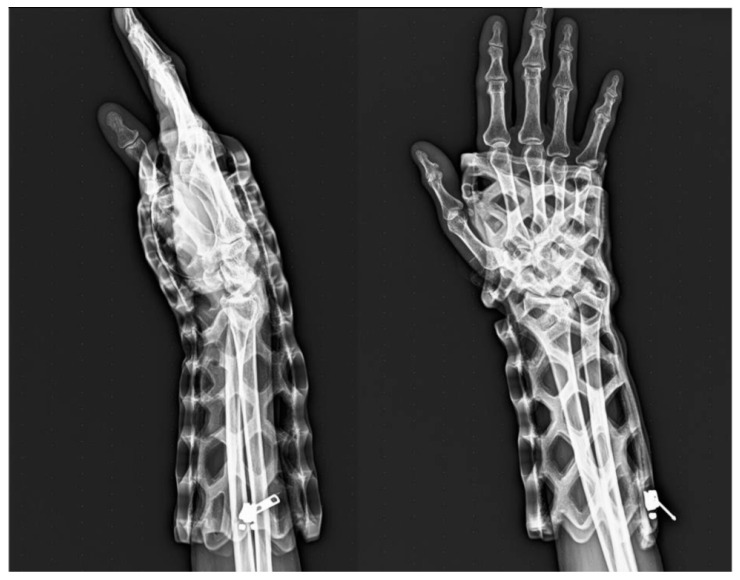
Radiographic image of the wrist while immobilized in a LCPI cast. The figure displays fracture alignment and visualization possible through the translucent immobilizer.

The light-cured polymer immobilization (LCPI) system used in this study consists of a photo-curable polymer lattice combined with internal padding and an external protective layer. The device is lightweight, breathable, and water-resistant and may be removed without the use of a traditional oscillating cast saw. The structural design and application of the device are illustrated in Figure 3. Radiographic visualization through the immobilizer is demonstrated in Figure 4.

### 2.5. Sample Size

A formal sample size calculation was not performed because of the retrospective observational design. Instead, all consecutive eligible patients treated during the study period were included to provide a clinically representative cohort. The final sample of 108 patients was considered sufficient to allow for descriptive estimation of healing outcomes and device-related events. Cohort sizes in the range of 80–150 patients have been reported in prior retrospective orthopaedic outcome studies evaluating fracture management and immobilization strategies [23,24].

### 2.6. Outcome Measures

Radiographic fracture healing was evaluated at the time of device removal and at the most recent clinical follow-up. Healing status was categorized based on treating clinician documentation as healed, partially healed, or not healed. Fracture alignment at follow-up was similarly classified as anatomic or near-anatomic, acceptable, or malaligned according to clinical and radiographic assessments recorded in the medical record (Table 2) [25]. These classifications were based on routine clinical documentation and were not independently standardized or adjudicated for study purposes.

Device-related and skin-related events documented during the period of immobilization were recorded, including breakage, irritation, or other soft-tissue concerns. Information regarding whether immobilization was completed for the intended duration or removed prematurely was also collected. When premature removal occurred, available documentation regarding timing, responsible party (patient or clinician), and clinical rationale was reviewed.

All outcome data were obtained from existing clinical records. No additional patient contact or imaging review was performed for study purposes. Consecutive case inclusion and the use of a standardized abstraction process were intended to reduce selection and information bias inherent to retrospective observational designs [26].

### 2.7. Statistical Analysis

Descriptive statistics were used to summarize demographic, clinical, and outcome variables [27]. Union rates were reported as percentages and 95% confidence intervals [28]. Continuous variables (age and healing time) were summarized as means with standard deviations or medians with interquartile ranges [29]. Categorical variables were presented as frequencies and percentages [30]. All analyses were conducted using IBM SPSS Statistics version 29.0 (IBM Corp., Armonk, NY, USA). Missing data were not imputed.

### 2.8. Ethical Approval

This study was reviewed and approved by the Kean University Institutional Review Board (Federal Wide Assurance #FWA00012551; IRB-FY2025-12). The requirement for informed consent was waived due to the retrospective design and use of de-identified clinical data. All study procedures were conducted in accordance with institutional policies and the ethical principles outlined in the Declaration of Helsinki [31].

## 3. Results

### 3.1. Patient Characteristics and Injury Distribution

**Figure 5:**
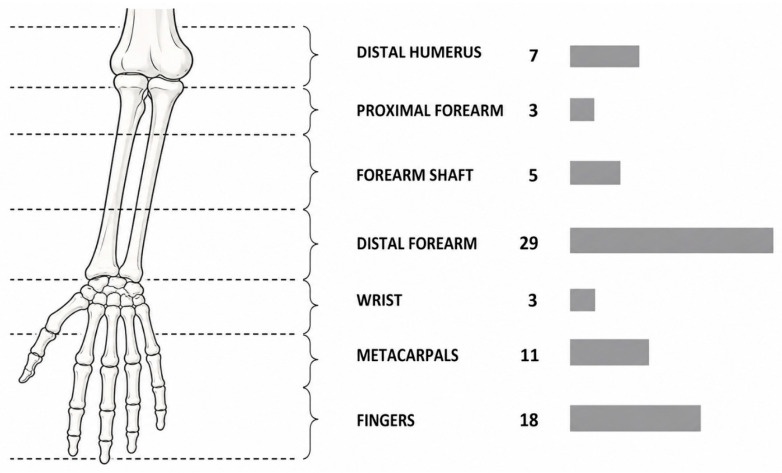

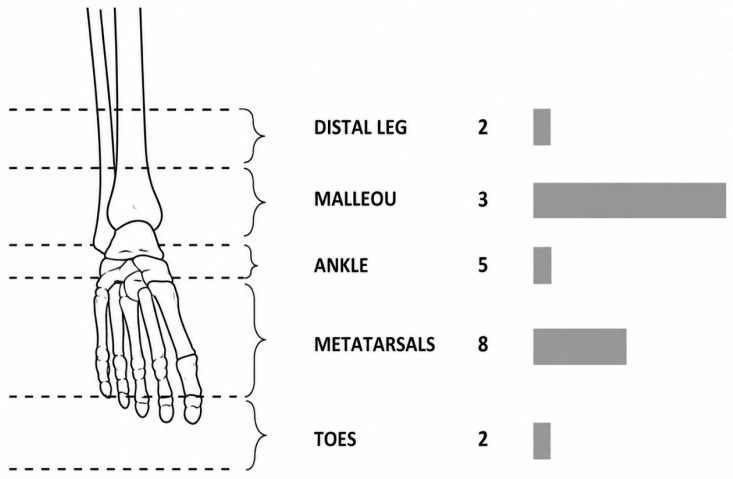
Anatomical distribution of fracture sites in the upper and lower extremities. Locations include distal forearm, metacarpals, phalanges, malleoli, metatarsals, and other defined injury regions.

Immobilization using an LCPI system was applied in 108 cases during the study period. Applications were predominantly for fracture management (104/108, 96.3%), with a small number of cases involving sprains (*n* = 3) and one elbow dislocation (Table 3). Among fracture cases, the majority were undisplaced (92/104, 88.5%) and were managed nonoperatively. A smaller proportion of fractures were displaced (11/104, 10.6%), including cases treated with closed reduction followed by immobilization and those managed postoperatively. The anatomical distribution of injuries is presented in Table 4 and Figure 5. Upper-extremity injuries accounted for 77 of 108 cases (71.3%), most commonly involving the distal forearm (*n* = 29), fingers (*n* = 18), and metacarpals (*n* = 11). Lower-extremity injuries represented 31 cases (28.7%), predominantly malleolar (*n* = 17) and metatarsal fractures (*n* = 8).

### 3.2. Initial Management Prior to Light-Cured Polymer Immobilization

Prior to application of the LCPI system, most patients received temporary immobilization as part of routine clinical care to allow for resolution of swelling. Initial management most commonly involved splinting (*n* = 94). A smaller number of patients were treated with interim fiberglass or plaster casting (*n* = 10) or a wrist brace (*n* = 1). Three patients underwent definitive immobilization without preceding temporary support. The mean duration of interim splint use was 5 days (interquartile range: 4 days; range: <1–31 days). Among patients who received temporary casting or bracing prior to definitive immobilization, duration ranged from 4 to 23 days.

### 3.3. Type of Light-Cured Polymer Immobilization Applied

Several immobilization configurations were used according to injury location and clinical indication (Table 5). The most frequently applied configurations were short leg (*n* = 32) and short arm (*n* = 29) constructs. Other applications included radial gutter (*n* = 15), long arm (*n* = 15), ulnar gutter (*n* = 11), and both long and short thumb spica constructs (*n* = 3 each).

### 3.4. Post-Removal Management

Following device removal, most patients (103/108) required no further immobilization. Five patients received additional support based on clinical findings at follow-up. Subsequent management included transition to walking boots after confirmation of fracture healing in two cases, application of a fiberglass cast in two cases related to device breakage or patient nonadherence, and temporary splinting in one case due to a documented skin reaction. Details of post-removal management are summarized in Table 6.

### 3.5. Duration of Immobilization

**Figure 6:**
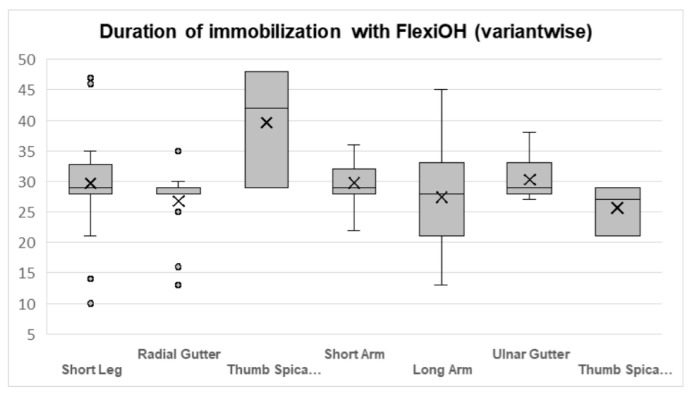
Duration of immobilization with the LCPI per variant. Box-and-whisker plot illustrating the distribution, median, range, and mean duration of immobilization for each FlexiOH variant, including Short Arm, Long Arm, Ulnar Gutter, Radial Gutter, Thumb Spica, and Short Leg immobilizers. The “X” within each box represents the mean value. Values reflect days in cast for all patients who completed the intended duration of treatment.

The duration of immobilization is summarized in Figure 6. The mean immobilization period across the cohort was 29.2 days (SD: 6.2; range: 10–48), with a median duration of 29 days. Duration varied modestly by immobilization configuration. Thumb spica constructs demonstrated the longest mean duration (32.7 ± 9.3 days), followed by long arm and short leg configurations (both 30.3 days), whereas radial and ulnar gutter configurations were associated with shorter immobilization periods (mean: 27.8 days).

### 3.6. Elective and Premature Cast Removal

The planned duration of immobilization was completed in 104 of 108 patients. Premature removal occurred in four cases (3.7%), all involving undisplaced fractures initially managed with short-term splinting prior to definitive immobilization. Documented reasons for early removal included device breakage (*n* = 3) and a skin reaction (*n* = 1). Following premature removal, patients were managed according to clinical findings at follow-up, including brief re-immobilization or observation. Subsequent radiographic assessments documented progression to fracture healing in all cases (Table 7).

### 3.7. Radiographic Healing and Alignment Outcomes

Radiographic healing outcomes were assessed in 104 patients treated for fracture management. Premature device removal occurred in four cases, leaving 100 patients who completed the intended duration of immobilization. Among these patients, radiographic union or advanced healing was documented in all cases completing the intended immobilization period at the time of device removal. No cases were recorded as partially healed or unhealed (Table 8). Postoperative immobilization was used in eight fracture cases, all of which demonstrated radiographic union in anatomic or near-anatomic alignment during follow-up. Across the full cohort of fracture cases, alignment at the time of device removal was classified as anatomic or near-anatomic in 103 patients (99.1%; 95% CI: 97.2–100) and acceptable in one patient. No documented loss of reduction requiring additional surgical or non-surgical intervention was identified.

### 3.8. Maintenance of Reduction and Skin-Related Events

Maintenance of fracture or joint reduction during immobilization was documented in five patients, including cases involving dislocation, fracture dislocation, and displaced or minimally displaced fractures (Table 9). Follow-up clinical documentation indicated progression to fracture healing with preservation of acceptable alignment in these cases. Skin-related adverse events were recorded in two of 108 patients (1.9%; 95% CI: 0.0–4.4) (Table 10). Reported findings included localized rash, erythema, and pruritus requiring device removal and temporary alternative immobilization. Both patients were managed with topical therapy and demonstrated resolution of symptoms during follow-up without documented long-term sequelae.

### 3.9. Adverse Events During Immobilization

Mechanical or structural device-related events were documented in 12 of 108 patients (11.1%; 95% CI: 5.2–17.0), all occurring in cases treated with nonoperative fracture immobilization (Table 11). Breakage was the only reported mechanical issue; no documentation of loosening, tightening, odor, or displacement was identified. Among patients with documented breakage, three removed the device prior to the intended duration of immobilization, whereas the remaining cases completed the planned immobilization period with breakage noted at follow-up assessment.

Distribution of breakage according to demographic and immobilization characteristics is presented in Table 12. Breakage was most frequently observed in patients aged 10–15 years (*n* = 6) and occurred more commonly in males (*n* = 9). With respect to immobilization configuration, ulnar gutter constructs accounted for the largest proportion of breakage events (*n* = 6), followed by short leg constructs (*n* = 3). A summary of key clinical and radiographic outcomes across study subgroups is provided in Table 13.

## 4. Discussion

### 4.1. Principal Findings

This retrospective cohort study described radiographic healing patterns, alignment outcomes, and documented adverse events among patients treated with an LCPI system in routine orthopaedic practice. Favorable healing progression and a low frequency of recorded skin- and device-related events were observed within a cohort consisting largely of pediatric patients with predominantly nondisplaced fractures managed nonoperatively. These findings should be interpreted strictly as descriptive of clinical outcomes observed in selected patients treated with LCPI and should not be interpreted as evidence of superiority or equivalence compared with conventional casting or other immobilization methods. Given the injury profile of the cohort, the observed healing patterns likely reflect, at least in part, the generally favorable prognosis of stable fractures in younger patients. This series contributes descriptive real-world data regarding clinical utilization of LCPI systems across a range of indications. The present findings are most applicable to low-risk fracture presentations, particularly nondisplaced injuries in pediatric and adolescent patients, and should be interpreted within this specific clinical context.

### 4.2. Interpretation in the Context of Existing Literature

Findings in the present cohort are generally consistent with previously reported observations involving light-cured polymer immobilization systems in similar patient populations [20,21]. No pressure sores, burns, or maceration were documented, and only a small number of minor skin-related findings were recorded. The absence of more severe complications, such as compartment syndrome, is not unexpected given that the cohort consisted predominantly of nondisplaced fractures managed nonoperatively. However, complication rates associated with fracture immobilization vary substantially across patient populations, injury severity, and treatment settings, with prior literature describing higher rates in selected contexts [9]. Radiographic healing and alignment outcomes were documented among patients completing the intended immobilization period, with immobilization durations broadly consistent with expected fracture healing timelines [32,33]. Because the present cohort consisted predominantly of nondisplaced fractures in children and adolescents, the favorable radiographic outcomes observed should not be interpreted as demonstrating comparative effectiveness of LCPI systems over conventional casting or other immobilization strategies.

### 4.3. Clinical Implications and Practical Considerations

Prior reports describing LCPI systems have documented their use in selected fracture populations, with generally favorable short-term clinical observations [20]. The present findings align with previously reported observations and contribute additional real-world data across a broader range of indications. Design characteristics such as reduced device weight, potential for ventilation, and removal without oscillating saw use may influence patient experience and clinical workflow; however, the relative importance of these factors has not been established in comparative trials [32,34]. Consideration of patient age, injury characteristics, resource availability, and clinician familiarity with different immobilization strategies remains important when selecting an appropriate treatment approach. In addition, the absence of a comparator group in the present study limits the ability to determine how these outcomes relate to conventional casting or alternative immobilization strategies.

Cost considerations are also important when evaluating the clinical utility of emerging immobilization technologies. Although the present study did not assess the cost of LCPI, how its use was financed within the clinical setting, or how these costs compare with conventional casting or brace-based management, differences in material cost, application time, follow-up requirements, and patient experience may all influence overall value. The relative cost-effectiveness of LCPI systems compared with traditional immobilization methods remains an important area for future investigation [35].

Closed reduction, when required, was performed prior to application of the LCPI system according to standard clinical practice. In these cases, fracture alignment was achieved using conventional reduction techniques before immobilization, and the LCPI system was then applied to maintain the corrected position. Procedural details such as sedation methods or reduction techniques were not consistently documented in the retrospective dataset and were therefore not analyzed. Given the material characteristics of LCPI systems, including the need for light-curing during application, their role in fractures requiring manipulation may be more limited compared with traditional casting techniques that allow for progressive molding. In the present cohort, only a small proportion of cases required reduction, and the findings should therefore be interpreted primarily within the context of nondisplaced fracture management.

Mechanical device breakage, observed in 11.1% of cases, was the most frequently documented device-related event in the present cohort. Although this rate may appear notable, breakage did not uniformly result in adverse clinical outcomes, and no cases of loss of reduction or major complication were attributed to device failure, as most patients were able to complete the intended immobilization period or were managed with minor adjustments in care. In this context, most breakage events can be considered clinically benign as they did not require intervention or alter the course of treatment, whereas a small subset (*n* = 3) required additional immobilization and could be considered clinically relevant mechanical failures. The clinical significance of this finding remains uncertain in the absence of a comparator group as rates of cast-related complications, treatment adjustments, and outcomes with conventional materials vary across studies and clinical settings [10,36]. Without direct comparison to plaster or fiberglass casting, the relative frequency and clinical importance of these events cannot be definitively established. These findings highlight the importance of further comparative investigation to determine whether breakage rates differ meaningfully between immobilization methods and how such events impact overall treatment effectiveness and patient experience.

### 4.4. Study Strengths and Limitations

This study has several strengths, including consecutive case inclusion, standardized retrospective data abstraction, and reliance on routine clinical documentation of radiographic healing and alignment. These features provide a descriptive overview of real-world clinical utilization patterns associated with LCPI use within a defined orthopaedic practice setting.

The most important limitation of this study is the absence of a comparison cohort, which precludes direct evaluation of LCPI systems relative to conventional immobilization methods. The retrospective single-center design limits control over potential confounding variables and precludes direct statistical comparison with conventional immobilization strategies such as plaster casting, fiberglass casting, or brace-based management, thereby limiting interpretation of relative effectiveness. The study cohort consisted predominantly of pediatric and adolescent patients, with the majority under 20 years of age, and largely involved nondisplaced fractures managed nonoperatively, which represents a key limitation, as these injury patterns are typically associated with favorable healing outcomes regardless of immobilization method. As such, the findings may not be directly generalizable to adult populations or to more complex, displaced, or unstable fracture patterns commonly encountered in broader orthopaedic practice. As a result, the observed outcomes cannot be attributed to the immobilization approach itself and may not be generalizable to displaced fractures or unstable injury patterns. A small number of non-fracture cases (sprains and one elbow dislocation) were included to reflect real-world clinical utilization; however, these represented a minor proportion of the cohort and are unlikely to have meaningfully influenced the overall findings, which are primarily driven by fracture outcomes.

Treatment selection was based on clinician judgment and evolving clinical circumstances, introducing the potential for selection bias, as LCPI systems may have been preferentially applied to patients with more stable fracture patterns or clinical characteristics associated with a lower risk of complications. In addition, variability in follow-up duration and reliance on treating clinician documentation rather than independent blinded radiographic assessment may have influenced outcome classification. This approach introduces the potential for measurement and interpretation bias as outcome classification was dependent on routine clinical documentation rather than standardized, independently adjudicated criteria. The study did not evaluate whether some fractures included in the cohort, particularly nondisplaced injuries, could have been successfully managed using less restrictive interventions such as removable braces or splints, which are commonly used in similar clinical scenarios. Nor did it include long-term follow-up to assess delayed union, refracture, functional recovery, or patient-reported outcomes. In addition, although differences in immobilization duration were observed across construct types, the relationship between immobilization duration and fracture type or anatomical location was not formally evaluated. These differences likely reflect underlying injury characteristics and clinical decision-making rather than device-specific factors and represent an area for future investigation.

Economic considerations were not examined. The relative cost of LCPI systems compared with traditional casting materials or brace-based treatment was not assessed, and differences in resource availability, regulatory approval, and institutional workflow requirements may influence broader implementation. Consequently, the present findings should be interpreted as descriptive and hypothesis-generating rather than evidence of comparative clinical effectiveness.

### 4.5. Future Directions

Future research should include prospective, controlled, and multicenter studies directly comparing LCPI systems with conventional casting and brace-based management to evaluate relative effectiveness, safety, and clinical outcomes across diverse patient populations. In addition, further investigation is needed to evaluate the relationship between fracture type, anatomical location, and optimal immobilization duration across different treatment approaches.

## 5. Conclusions

In this retrospective cohort, patients treated with an LCPI system were documented to have radiographic healing progression consistent with expected clinical patterns and maintenance of satisfactory alignment, with a low frequency of documented complications. These findings should be interpreted as descriptive radiographic healing and maintenance of acceptable alignment outcomes observed in a predominantly pediatric cohort with largely nondisplaced fractures rather than as evidence of comparative effectiveness of LCPI systems. Radiographic progression to union and maintenance of satisfactory alignment were observed in fracture cases completing the intended period of immobilization. A small number of minor skin-related events were recorded and resolved with conservative management. These findings are generally consistent with previously reported clinical experiences involving light-cured polymer immobilization systems. Further prospective, controlled, and multicenter investigations are warranted to better characterize healing outcomes, complication patterns, long-term safety, and potential economic considerations.

## Figures and Tables

**Table 1:** Sex Distribution of the Study Population (N = 108).

Sex	Number of Patients
Male	76
Female	32
Total	108

**Table 2:** Operational Definitions of Displacement, Healing Status, and Alignment.

Displacement	Operational Definition
Undisplaced	-
Minimally displaced	≤2 mm and ≤10°
Displaced	>2 mm and >10°
**Fracture healing**	
Not Healed	No evidence of healing
Partially Healed	Evidence of early callus or cortical bridging in ≤2 cortices; fracture line still visible; (reduced pain, not full function)
Healed	Radiographic bridging in ≥3 cortices; fracture line may persist faintly; painless at rest + light use.
**Fracture alignment**	
Anatomic or Near-Anatomic	Alignment within normal or near-normal limits (≤5° angulation/rotation, ≤5 mm shortening/translation).
Acceptable Alignment	Mild malalignment (5–10° angulation, 5–10 mm shortening); still within functional thresholds.
Malalignment (Functional)	Moderate malalignment (>10° or >10 mm), but does not cause functional impairment or symptoms.
Gross Malalignment	Severe misalignment, functionally limiting or requiring surgical correction.

Note: Bolded text indicates major outcome categories used as section headers within the table.

**Table 3:** Indications for Light-cured Polymer Immobilizer Application.

Diagnosis	*n*	Management (Conservative/Surgical)	Manual Reduction Performed	Indication for Light-Cured Polymer Immobilizer Application
**Fractures**	**104**			
Non-displaced	92	Conservative (*n* = 92)	0	Fracture immobilization
Minimally displaced	1	Conservative (*n* = 1)	0	Fracture immobilization
Displaced	11	Surgical (*n* = 8)/Conservative (*n* = 3)	3 **	Post-surgical immobilization/fracture immobilization
**Sprains**	3	Conservative	0	Immobilization post sprain
**Dislocations**	1	Conservative	1 **	Immobilization post elbow dislocation
Total	108			

** Post casting check X-rays showed adequate reduction achieved in all these patients. Note: Bold formatting denotes major diagnostic category groupings within the table.

**Table 4:** Anatomical Distribution of Injuries Treated.

Region	Anatomical Site	*n*		
	Upper Limb		Lower Limb	
	Distal humerus	7	Distal leg	2
	Elbow (joint)	1	Malleolar	17
	Proximal radius	2	Ankle	2
	Proximal ulna	1	Metatarsal	8
	Forearm—shaft	5	Toe	2
	Forearm—distal	29		
	Wrist	3		
	Metacarpal	11		
	Finger	18		
	Subtotal	108		

**Table 5:** Distribution of Light-cured Polymer Immobilizer Cast Variants Used.

Variant Name	*n*
Short Leg	32
Short Arm	29
Radial Gutter	15
Long Arm	15
Ulnar Gutter	11
Thumb Spica (Long)	3
Thumb Spica (Short)	3
Total	108

**Table 6:** Treatment after Light-cured Polymer Immobilizer (LCPI).

Device Used	*n*	Reason
None	103	Further immobilization not needed
Fiberglass Thumb Spica	1	LCPI Broke on day 13
Walking boot	2	Post casting support
Fiberglass Short leg cast	1	LCPI Broke on day 14
Fiberglass Short Arm Splint	1	LCPI removed due to skin rash
Total	108	

**Table 7:** Summary of cases with premature LCPI removal (*n* = 4).

Variable	Summary
Age range	10–20 years
Sex	3 male, 1 female
Injury types	Phalangeal fractures (*n* = 2), distal radius torus fractures (*n* = 2)
Immobilization types	Radial gutter (*n* = 1), short arm (*n* = 2), short leg (*n* = 1)
Time to removal	10–28 days
Reasons for removal	Cast breakage (*n* = 3), skin irritation (*n* = 1)
Radiographic status at removal	Partially healed (*n* = 3), fully healed (*n* = 1)
Post-removal management	Additional immobilization (*n* = 2), no further treatment (*n* = 2)
Final outcome	100% fracture union

**Table 8:** Radiographic Healing Outcomes at elective Light-cured Polymer Immobilizer Removal by Treatment Type.

Treatment Type	Fully Healed	Healed	Partially Healed	No Healing	Total
Conservative fracture immobilization	91	1	0	0	92
Post-surgical support	8	0	0	0	8
Total	99	1	0	0	100

**Table 9:** Summary of Reduction Stability and Healing Observations.

Case	Diagnosis/Injury Type	Light-Cured Polymer Immobilizer Variant	Reduction Status	Duration of Immobilization (Days)	Healing Outcome	Notable Findings
Dislocation						
1	Pathological elbow dislocation	Long Arm	Maintained	13	Concentric reduction maintained	No complications
Fracture dislocation						
2	Middle phalanx fracture with PIP dislocation	Ulnar Gutter	Maintained	28	Fully Healed in anatomic/near anatomic alignment, with PIP reduction maintained	Minor cast breakage ^1^
Fractures						
1	Displaced right scaphoid fracture	Short Arm	Maintained	48 ^2^	Fully Healed in anatomic/near anatomic alignment	No complications
2	Displaced distal radius fracture	Short Arm	Maintained	31	Fully Healed in anatomic/near anatomic alignment	No complications
3	Minimally displaced R Lateral Malleolus fracture	Short Leg	Maintained	30	Fully Healed in anatomic/near anatomic alignment	No complications

^1^ incidental finding at cast removal. ^2^ Extended duration due to late-presenting displaced scaphoid fracture; not device-related.

**Table 10:** Summary of Skin-Related and Clinical Adverse Events.

Clinical Adverse Event	*n*
None	106
Erythema (No rash, itching or blisters)	0
Rash with itching	1
Rash with itching, vesicles, blisters, and exudate	1
Pressure sore	0
Ulcer	0
Compartment syndrome	0
Other (specify)	0

**Table 11:** Summary of Cast-Related Events.

Cast-Related Event	*n*
None	96
Cast breakage	12
Foam cracking	0
Foul smell	0
Cast loosened over time	0
Cast tightened over time	0
Silicone cover torn	0
Foam pieces displaced	0
Foam pieces lost	0
Other	0

**Table 12:** Characteristics of Patients with Cast Breakage (*n* = 12).

Age Group (Years)	*n*	Sex	*n*	FlexiOH Variant	*n*
<5	0	Male	9	Ulnar Gutter	6
5–<10	2	Female	3	Short Leg	3
10–<15	6			Radial Gutter	1
15–20	4			Long Arm	1
				Short Arm	1
Total	12	Total	12	Total	12

**Table 13:** Summary of Clinical Outcomes Across the Light-cured Polymer Immobilizer Cohort (N = 108).

Domain	Finding
Total Cohort	108 immobilizations
Primary Indications	104 fractures (96.3%), 3 sprains (2.8%), 1 elbow dislocation (0.9%)
Treatment Type (Fractures)	96 conservative, 8 post-surgical
Fracture Characteristics (Conservative Group)	92 non-displaced; 1 minimally displaced; 2 displaced; 1 fracture-dislocation
Alignment at Final Follow-up (Conservative Group)	95/96 anatomic/near-anatomic; 1/96 acceptable
Bone Healing Outcome (All Fractures)	103/104 fully healed; 1/104 healed (lost to follow-up after week 4)
Elective vs. Premature Removal (All Casts)	104 elective removals; 4 premature (3 breakage, 1 rash)
Healing Status at Premature Removal (*n* = 4)	3 partially healed; 1 fully healed
Post-Removal Management (Premature Removals)	2 no further immobilization; 2 transitioned to fiberglass/splint
Soft-Tissue Injuries (*n* = 4)	3 sprains resolved uneventfully; 1 pathologic elbow dislocation maintained reduction
Skin-related Adverse Events	2/108 rashes; all resolved with topical treatment (2–3 weeks)
Cast-related Adverse Events	12/108 breakages (3 affecting course of treatment; 9 incidentals at removal)

## Data Availability

The datasets analyzed during the current study are not publicly available due to institutional and ethical restrictions but may be available from the corresponding author on reasonable request.

## Supplementary Materials

The following supporting information can be downloaded at: https://www.mdpi.com/article/10.3390/jcm15103709/s1, Table S1: STROBE Statement—Checklist of items that should be included in reports of *cohort studies*.

## Abbreviations

The following abbreviations are used in this manuscript:

LCPI	Light-Cured Polymer Immobilizer/Immobilization
POP	Plaster of Paris
ORIF	Open Reduction and Internal Fixation
CI	Confidence Interval
